# Association of SCARB1 Gene Polymorphisms with Virological Response in Chronic Hepatitis C Patients Receiving Pegylated Interferon plus Ribavirin Therapy

**DOI:** 10.1038/srep32303

**Published:** 2016-08-26

**Authors:** Ching-Sheng Hsu, Shih-Jer Hsu, Wei-Liang Liu, Ding-Shinn Chen, Jia-Horng Kao

**Affiliations:** 1 Division of Gastroenterology, Department of Internal Medicine, Taipei Tzu Chi Hospital, Buddhist Tzu Chi Medical Foundation, Taipei, Taiwan; 2 School of Post-Baccalaureate Chinese Medicine, Tzu Chi University, Hualien, Taiwan; 3 School of Medicine, Tzu Chi University, Hualien, Taiwan; 4 Department of Internal Medicine, National Taiwan University Hospital, Yun-Lin Branch, Yun-Lin County, Taiwan; 5 Graduate Institute of Clinical Medicine, National Taiwan University College of Medicine, Taipei, Taiwan; 6 Department of Internal Medicine, National Taiwan University College of Medicine and National Taiwan University Hospital, Taipei, Taiwan; 7 Department of Medical Research, National Taiwan University College of Medicine and National Taiwan University Hospital, Taipei, Taiwan; 8 Hepatitis Research Center, National Taiwan University College of Medicine and National Taiwan University Hospital, Taipei, Taiwan

## Abstract

The scavenger receptor type B class I(SR-BI) is a receptor for high-density lipoproteins(HDL) and one of entry factors for hepatitis C virus(HCV). We examined the association of single nucleotide polymorphisms(SNPs) of the SCARB1 gene, which encodes SR-BI, with virologic responses to pegylated interferon-based treatment in Asian chronic hepatitis C(CHC) patients. Human genomic and clinical data were collected from 156 consecutive Taiwanese HCV genotype 1 or 2 patients who received pegylated interferon plus ribavirin therapy and 153 non-HCV healthy subjects. Three SNPs(rs10846744, rs5888, and rs3782287) of the SCARB1 gene that have been linked to humans diseases were investigated. rs10846744 rather than rs5888 or rs3782287 was associated with serum HCV RNA level and sustained virologic response(SVR) to pegylated interferon plus ribavirin therapy in CHC patients(GG vs. non-GG genotype, Adjusted Odds Ratio, 95% CI: 0.32, 0.11–0.95, P = 0.039). Among patients with IL28B rs8099917 non-TT genotypes, those with rs10846744 non-GG genotype had a higher SVR rate than those with GG genotypes. In addition, patients with GG genotype had a higher fasting blood glucose level than those with CC genotype. In conclusion, SCARB1 gene polymorphisms may serve as a potential predictor of treatment responses in CHC patients receiving interferon-based therapy. (ClinicalTrials.gov number, NCT02714712).

Hepatitis C virus (HCV) infection is the leading cause of liver diseases, hepatocellular carcinoma and liver transplantation worldwide^1^,^2^. Although several HCV-specific, direct acting antivirals (DAAs) that targeting viral replication and assembly have been approved for the treatment of chronic hepatitis C(CHC) patients, these regimens remain expensive and most hepatitis C patients in developing countries cannot afford them^3^,^4^. Thus, interferon (IFN)-based therapy is still used as the standard care of HCV infection in many parts of the world^5^,^6^.

Chronic HCV infection is well-known for its interaction with host lipid metabolism. Infectious HCV particles and serum lipoproteins may form hybrid “lipoviral particles” (LVPs) that can facilitate its interaction with viral entry factors and send HCV particles into cells^7^,^8^. Among these entry factors, the scavenger receptor class B type I (SR-BI) protein, which is highly expressed on hepatocytes, encoded by the SCARB1 gene, and a receptor for high density lipoproteins (HDL), very-low-density lipoproteins (VLDL) and oxidized forms of LDL^9^, may interact with virus-associated lipoproteins to help the attachment and binding of HCV particles^10^. Of note, several studies have reported the associations of SCARB1 single nucleotide polymorphisms (SNPs) with serum HDL, LDL levels^11^,^12^,^13^,^14^,^15^, and various human diseases^16^,^17^,^18^,^19^. However, although SR-BI protein plays an essential role in HCV infection, the impact of SCARB1 gene SNPs on HCV infection remain largely unknown.

In this study, we investigated the relationship of SCARB1 gene SNPs with HCV infection by comparing the human genomic and clinical data between CHC patients receiving pegylated interferon alfa-2a plus ribavirin (PR) therapy and non-HCV controls. Moreover, we also examined the association of these SNPs with treatment responses and metabolic profiles in Asian CHC patients.

## Results

### Baseline characteristics of study population

A total of 309 subjects with available human genomic DNA were enrolled, including 156 patients with chronic HCV genotype 1 or 2 infection and 153 non-HCV controls. Among CHC patients, 110(70.5%) patients attained rapid virologic response (RVR) to pegylated interferon alfa-2a (Peg-IFN alfa-2a) plus ribavirin therapy and 118 (75.6%) achieved SVR. The distributions of baseline demographic and metabolic characters are shown in Table 1. In brief, patients in the HCV group had significantly higher body mass index (HCV vs. Control, mean ± SD, kg/m^2^: 25.4 ± 3.7 vs. 23.8 ± 3.5) and serum alanine aminotransferase (ALT) level (U/L: 138.0 ± 120.8 vs. 27.0 ± 16.2), but lower platelet counts (K/ul: 174.2 ± 54.9 vs. 249.5 ± 62.0) than subjects in the control group. Compared to non-HCV controls, CHC patients had a significantly higher fasting blood glucose level (mg/dL: 106.1 ± 29.0 vs. 96.1 ± 14.6), but lower serum total cholesterol level (mg/dL: 171.9 ± 34.5 vs. 198.3 ± 34.6), HDL (mg/dL: 45.1 ± 11.6 vs. 56.1 ± 17.4) and LDL levels (mg/dL: 101.0 ± 31.6 vs. 127.8 ± 32.3).

### Association of SCARB1 genotypes with metabolic profiles

The frequencies of SCARB1 and IL28B genotypes were in Hardy-Weinberg Equilibrium in the whole study population (P = 0.4677 for rs10846744 genotype, P = 0.8527 for rs5888 genotype, and P = 0.1484 for rs3782287). Because SR-BI protein is a receptor for lipoproteins and plays an essential role in host metabolism, we hypothesized that SCARB1 genotype may affect serum metabolic profiles in the study population. We examined the relationship of SCARB1 genotypes and metabolic profiles in the whole study population, HCV and control groups, and found that patients with rs10846744 GG genotype had comparable serum triglyceride, total cholesterol, LDL and HDL levels to those with CC genotype. However, patients with rs10846744 GG genotype had higher fasting blood glucose levels than those with CC genotype (mean ± SD, mg/dL: 108.2 ± 28.4 vs. 100.7 ± 24.8, P = 0.044). Of note, this difference was significant only in patients with chronic HCV infection (mg/dL: 112.2 ± 35.3 vs. 100.5 ± 20.4, P = 0.045), but not in controls (Table 2). However, patients with rs5888 or rs3782287 AA/GA genotype had comparable serum triglyceride, total cholesterol, LDL, HDL and fasting blood glucose levels to those with GG genotype in the whole study population, HCV and control groups.

### Association of SCARB1 genotypes with serum HCV RNA level

As SR-BI protein not only interacts with virus-associated lipoproteins but also helps the attachment and binding of HCV particles, we hypothesized that SCARB1 SNPs may affect HCV replication and serum HCV RNA level as well. We thus examined the association of SCARB1 genotype and serum HCV RNA level among CHC patients and found that patients with rs10846744 GG genotype had significantly lower serum HCV RNA levels than the ones with CC genotype (IU/mL: 5.68 ± 1.05 vs. 6.10 ± 0.98, P < 0.05) (Table 3).

### Association of SCARB1 genotypes with virological responses

In this study, patients with favorable IL28B genotype (rs8099917 TT genotype) had higher RVR, early virologic response (EVR) and SVR rates than those with GT genotype (P < 0.05). Although patients with rs5888 and rs3782287 GG genotype had comparable RVR, EVR and SVR rates to those with AG or AA genotype, those with rs10846744 CC/CG genotype had a higher SVR rate than those with GG genotype (P = 0.028) (Table 4). This difference remained significant after adjustment for age, body mass index (BMI), and ALT levels (P < 0.001). In multivariate analyses, rs10846744 GG genotype was significantly associated with a lower SVR in CHC patients (Adjusted Odds Ratio, 95% CI: 0.32, 0.11–0.95, P = 0.039) (Table 5). Moreover, among patients with unfavorable IL28B genotype (rs8099917 GT genotype), rs10846744 CC/CG genotype was associated with a higher SVR rate than GG genotype (P = 0.007) (Table 4).

Because gender, HCV genotype and treatment duration may modify the association of SCARB1 genotypes and SVR, we made sub-analyses stratified by these characters and found that rs10846744 CC/CG genotype was associated with a higher SVR rate in males, but not in females. The difference remained significant after adjustment for age and BMI (Adjusted Odds Ratio, 95% CI: 3.78, 1.05–13.6, P = 0.041). Stratifying the analyses by HCV genotype, we found that HCV genotype 1 patients with rs10846744 CC/CG genotype had a higher SVR rate than those with GG genotype (P = 0.021), but this fact was not observed in HCV genotype 2 or 3 patients (Supplementary Table S1). The difference remained significant after adjustment for age and BMI (Adjusted Odds Ratio, 95% CI: 2.88, 1.01–8.26, P = 0.048). Further stratifying the analyses by the duration of pegylated interferon plus ribavirin treatment, patients with rs10846744 CC/CG genotype had a higher SVR rate than those with GG genotype with a treatment duration ≤24 weeks, but it was not seen in patients receiving a treatment duration >24 weeks. The difference remained significant after adjustment for age and BMI (Adjusted Odds Ratio, 95% CI: 4.83, 1.30–18.0, P = 0.019).

## Discussion

Our data showed that SCARB1 rs10846744 GG genotype may serve as an unfavorable therapeutic factor for CHC patients receiving pegylated interferon plus ribavirin therapy. Compared to rs10846744 CC/CG genotype, GG genotype was associated with a decreased SVR rate and the Odds Ratio after adjusting for known prognostic factors was 0.32. Moreover, among the patients with unfavorable IL28B genotype and rs10846744 GG genotype, none could achieve SVR after pegylated interferon plus ribavirin treatment. In considering the key role of SR-BI in the entry for HCV particles, our data not only indicate the predictive values of SCARB1 genotype for CHC patients with interferon-based therapy but also imply a possible extrapolation of this predictor to those who receive interferon-free DAAs therapy.

Although SR-BI is well-known for its critical role on cholesterol metabolism^9^ and HCV entry^10^, its interactive pathways and functional mechanisms remain to be completely elucidated. In this study, patients with SR-BI rs10846744 GG genotype are likely to have both lower serum HCV RNA levels and poor SVR to anti-HCV therapy. This finding seems unexpectedly contradictory to the general concept that a lower serum HCV RNA level is usually associated with a better SVR to anti-viral therpay^20^,^21^. However, recent studies have shown the regulatory roles of SR-BI on lymphocyte homeostasis, including in the modulation of lymphocytes proliferation and cytokine production^22^, implying that SR-BI may not only affect HCV attachment/entry in hepatocytes, but also the response to anti-HCV therapy. Therefore, rs10846744 GG genotype may probably impede HCV attachment/entry in hepatocytes, leading to the decline of HCV replication and serum HCV RNA levels, and also impair the antiviral treatment, resulting in a lower therapeutic response and SVR rate in CHC patients.

We examined three SNPs (rs10846744, rs5888, and rs3782287) of the SCARB1 gene known to be associated with human diseases^13^,^16^,^17^,^18^,^19^. However, only the rs10846744 variant was identified as a predictor of virological response in CHC patients receiving treatment. Because rs10846744 GG genotype was associated with a lower SVR rate, but comparable RVR and EVR rates to those with CC or CG genotype, our data implied that rs10846744 variant may affect the relapse of treated CHC patients. As patients who relapsed are usually associated with treatment-related side effects, emotional stain, much healthcare expenditure, and significant decreases in measures of general health status^23^, information of rs10846744 genotype may help optimize the management of potential relapsers.

The rs10846744 variant has been recognized as a major predictor of subclinical atherosclerosis and incident cardiovascular diseases in the Multi-Ethnic Study of Atherosclerosis (MESA)^15^. It is located within the intron 1 of SCARB1 gene, containing DNase I hypersensitivity clusters, enhancer-promoter histone markers, and has no correlation with levels of SR-BI RNA or protein^16^. The rs10846744 variant was thus considered to exert cis or trans regulatory effects, or act as an enhancer, and possibly affected or altered expression of a distant gene^15^. Because the effect of SR-BI protein was independent of HDL-C levels^24^, the non-lipid pathways, such as affecting endothelial function^25^ or inflammatory pathways^26^, have been hypothesized to be the functional mechanisms of rs10846744 variant. Interestingly, recent studies also demonstrated an interaction between glucose metabolism and the expression of SR-BI^27^,^28^ through a transcriptional regulatory mechanism^29^,^30^. In comparison with the euglycemic control rats, administration of glucose could significantly decrease the hepatic expression of SR-BI in diabetic rats^29^,^30^. In our study, we found that the rs10846744 GG genotype was significantly associated with a higher fasting blood glucose level in subjects with HCV infection, but not in controls. This HCV-specific link between SCARB1 genotype and glucose metabolism implies that glucose metabolism may play a role in functional mechanisms of rs10846744 variant. Of note, HCV replication may interact with host glucose metabolism^31^, and improvement of metabolic profiles has been found to be associated with viral load decline^21^ and viral kinetic parameters^20^ in hepatitis C patients. Considering the link between rs10846744 variant, host metabolism, and HCV viral kinetics^20^,^21^,^32^,^33^, our data implied that the rs10846744 variant may exert its effect on HCV replication partly through the pathways of glucose metabolism to affect serum HCV RNA levels and treatment outcomes in CHC patients.

Our study had a few limitations. First, this retrospective study was a case-control design, and hence, only associations between chronic HCV infection and SCARB1 genotype could be determined. Studies with a longitudinal design, more following data and paired controls are required to explore the impact of disease stages (such as fibrosis, or cirrhosis, or hepatocellular carcinoma) on the association of SCARB1 genotype with HCV infection. Second, the rs10846744 variant was helpful in predicting virological response for specific subgroup populations, such as males, in patients with HCV genotype 1 infection, or receiving an interferon-based treatment duration ≤24 weeks. Future studies to explore its use in combination with other pretreatment clinical profiles and on-treatment viral kinetics are needed for the development of personalized HCV treatments^34^. Third, because the present study was based on patients received interferon-based treatment, further studies to verify its usefulness in patients receiving interferon-free DAAs regimens are needed. Finally, although the pathways of glucose metabolism may probably contribute to the association of the rs10846744 variant and HCV infection, more studies are required to examine the effects and mechanisms of this variant on each step of HCV life cycle.

In summary, the rs10846744 variant may serve as a potential predictor of the treatment responses in CHC patients with interferon-based therapy. Although the rs10846744 variant is associated with fasting blood glucose level in CHC patients, whether this association alters HCV entry and release of viral particles through metabolic regulation awaits further studies.

## Patients and Methods

### HCV group

A total of 156 chronic HCV genotype 1 or 2 patients who received PR therapy and were consecutively enrolled from the gastroenterological clinics of the National Taiwan University Hospital and its Yun-Lin branch for a previous viral kinetic study of “IL28B genotypes and metabolic profiles” were selected^20^. In brief, chronic HCV infection was defined as the positivity of both anti-HCV and serum HCV RNA for ≥6 months. All patients had serum ALT levels, at least, twice the upper limit of normal on two occasions within the previous 6 months. None of them had received IFN treatment, other experimental antiviral, or immunosuppressive therapy before enrollment, or were positive for hepatitis B surface antigen (HBsAg), human immunodeficiency virus (HIV) antibody, or had a known history, or serological evidence of autoimmune liver disease, inheritable disorders such as hemochromatosis or Wilson’s disease, renal insufficiency, malignancy, a history of daily alcohol consumption greater than 20 gram or active drug abuse.

All patients were treated with a weekly injection of Peg-IFN alfa-2a 180 μg plus daily oral weight-based ribavirin (1,000 mg for body weight ≤75 kg, 1,200 mg for body weight >75 kg) for 24 or 48 weeks based on the status of RVR^5^,^34^, and were followed for additional 24 weeks after the discontinuation of treatment.

### Control group

There were 153 healthy controls negative for anti-HCV enrolled simultaneously from the database of Health Management Center at the Taipei Tzu Chi Hospital, Buddhist Tzu Chi Medical Foundation between 2006 and 2007 as previously described^20^. We excluded subjects who had positive for HBsAg, HIV or those with insufficient information regarding HBsAg, anti-HCV, and HIV. Random selection without replacement was used to ensure that no control subject was assigned to more than once.

The primary outcomes of interest were the association of SCARB1 gene SNPs with SVR and metabolic profiles between CHC patients and non-HCV controls.

### Ethical considerations

The study was conducted in accordance with the principles of the Declaration of Helsinki and approved by the Ethical Committee of the National Taiwan University Hospital and Taipei Tzu Chi Hospital, Buddhist Tzu Chi Medical Foundation. All patients gave informed consents before enrollment. We recorded their viral parameters, biochemical, serologic as well as anthropometric data at enrollment.

### Definitions of treatment response

The virologic response to therapy was based on serum HCV RNA level. RVR was defined if an undetectable serum HCV RNA level at week 4 was achieved. EVR was defined as undetectable serum HCV RNA level or at least 2 log decrease of the baseline HCV RNA level at week 12. Complete early virologic response (cEVR) was defined as undetectable serum HCV RNA at week 12 of therapy, and partial early virologic response (pEVR) as an, at least, 2-log reduction of serum HCV RNA from baseline to week 12 of therapy. Relapse was defined as undetectable serum HCV RNA level at the end of treatment, but detectable serum HCV RNA level during follow-up. Sustained virologic responders were defined as those having undetectable serum HCV RNA 24 weeks after cessation of the treatment^21^,^35^.

### Baseline demographic and clinical features

We collected information such as gender, age, BMI, complete blood count, serum aspartate aminotransferase (AST), and alanine aminotransferase (ALT) levels. BMI was calculated as weight in kilograms divided by height in square meters. Blood samples were collected in the morning after 12 hours fasting and measured by standard laboratory techniques. Serum AST and ALT levels were measured by an autoanalyzer (Hitachi 7250, Special; Hitachi, Tokyo, Japan) according to the manufacturer’s instructions. The upper limit of normal (ULN) of serum ALT level was set at 30 U/L for men and 19 U/L for women^36^,^37^.

### Serological markers and Quantification of HCV RNA level

HBsAg and anti-HCV were assayed with commercial kits (Abbott Laboratories, North Chicago, IL, USA). HCV RNA level was determined by using the real-time PCR-based single-tube assay as previously described^38^,^39^.

### Extraction, quantification, and genotyping of HCV RNA

Serum HCV RNA was extracted by using a commercial kit (QIAamp RNA Blood Mini Kit; Qiagen Inc, Valencia, CA, USA), and quantified by the LightCycler (Roche Diagnostics Applied Science, Penzberg, Germany) with the detection limit of 86 copies/mL (i.e. 34 IU/mL)^40^. HCV genotyping was performed by the LightCycler PCR assay or reverse transcription-PCR (RT-PCR) with type-specific primers as previously described^41^,^42^. The detection limit of type-specific primers genotyping method is 100 copies/mL (i.e. 37 IU/mL). All samples were tested in triplicate.

### Extraction of human genomic DNA and genotyping of rs10846744, rs5888, rs3782287 and rs8099917

Three SNPs (rs10846744, rs5888, and rs3782287) of the SCARB1 gene with an allele minor frequency of >1% and have been linked to humans diseases were investigated^13^,^16^,^17^,^18^,^19^.

All enrolled subjects were genotyped for the SNPs of the SCARB1 gene (rs10846744, rs5888, and rs3782287) and IL28B gene (rs8099917) by using the ABI TaqMan allelic discrimination kit and the ABI7900HT Sequence Detection System (Applied Biosystems, Foster City, CA, USA)^43^. All their blood specimens were collected into EDTA tubes, and human genomic DNA was extracted by standard protocols, including blood RBC lysis, cell lysis, DNA binding, wash, and elution. Extracted DNA was normalized to 50 ng/μl, and assessed by calculating the absorbance ratio OD260 nm/280 nm using NanoDrop model ND-1000 (Thermo Scientific, Wilmington, DE, USA).

### Statistical analysis

Categorical data were presented as percentages while continuous data were presented as mean with standard deviations. Log transformation was performed for variables with a significant deviation from a normal distribution. Chi-square tests, t-tests, and Wilcoxon’s tests were used to analyze categorical, parametric continuous and non-parametric variables, respectively. Linear regression and multivariate analyses using logistic regression were performed to examine the associations of SNPs, SVR, and various clinical characters.

All analyses were performed with Stata statistical software (version 8.0, Stata corp., College Station, Tex). All tests were 2-sided and *P* < 0.05 was considered statistically significant.

## Additional Information

**How to cite this article**: Hsu, C.-S. *et al*. Association of SCARB1 Gene Polymorphisms with Virological Response in Chronic Hepatitis C Patients Receiving Pegylated Interferon plus Ribavirin Therapy. *Sci. Rep.* **6**, 32303; doi: 10.1038/srep32303 (2016).

## Supplementary Material

Supplementary Information

## Figures and Tables

**Table 1:** Comparisons of demographic and metabolic characteristics between chronic hepatitis C patients and non-HCV controls.

	HCV patients N = 156	Non-HCV controls N = 153	*P* value
Age, years	55.8 ± 10.0	52.4 ± 9.7	0.002
Male, %	89(57.1)	74(48.4)	0.126
BMI, kg/m^2^	25.4 ± 3.7	23.8 ± 3.5	<0.001
AST,U/L	94.9 ± 70.1	24.7 ± 9.0	<0.001
ALT, U/L	138.0 ± 120.8	27.0 ± 16.2	<0.001
PLT, K/ul	174.2 ± 54.9	249.5 ± 62.0	<0.001
Fasting blood glucose, mg/dL	106.1 ± 29.0	96.1 ± 14.6	<0.001
Triglyceride, mg/dL	113.8 ± 74.5	115.5 ± 66.2	0.827
Total cholesterol, mg/dL	171.9 ± 34.5	198.3 ± 34.6	<0.001
LDL, mg/dL	101.0 ± 31.6	127.8 ± 32.3	<0.001
HDL, mg/dL	45.1 ± 11.6	56.1 ± 17.4	<0.001
rs10846744 CC/CG/GG genotype, %	59(37.8)/72(46.2)/25(16.0)	46(30.3)/79(52.0)/27(17.8)	0.375
rs5888 GG/AG/AA genotype, %	86(55.5)/59(38.1)/10(6.5)	90(58.8)/54(35.3)/9(5.9)	0.839
rs3782287 GG/AG/AA genotype, %	103(66.5)/48(31.0)/4(2.6)	91(59.5)/50(32.7)/12(7.8)	0.092
HCV RNA Log_10_ IU/mL	5.8 ± 1.0	—	—
HCV Genotype 1, %	132(84.6)	—	
Peg-IFN duration >24 wk, %	51(32.7)		
SVR/non-SVR, n (%)	118/38(75.6/24.5)		
RVR/non-RVR, n(%)	110/46(70.5/29.5)		
cEVR/pEVR/non-cEVR, n(%)	134/10/4(90.5/6.8/2.7)		
EOTR, n(%)	150/4(97.4/2.6)		
APRI	1.59 ± 1.32	0.27 ± 0.19	<0.001
FIB-4	3.05 ± 1.97	1.14 ± 0.67	<0.001
METAVIR Fibrosis(F0-2/F3-4), n	45/34		

**Note.** Data is shown by mean ± standard error or case number (proportion).

Histologic data were evaluated semi-quantitatively using Metavir scoring system.

**Abbreviations:** BMI, body mass index; ALT, alanine aminotransferase; AST, aspartate aminotransferase; PLT, platelet count; Peg-IFN; pegylated interferon; SVR, sustained virologic response; RVR, rapid virologic response; EVR, early virologic response; cEVR, complete early virologic response; pEVR, partial early virologic response; EOTR, end-of-treatment response.

**Table 2:** Associations of metabolic profiles and SCARB1 genotypes in 156 chronic hepatitis C patients.

	rs10846744			*P* value
	CC genotype	CG genotype	GG genotype	
Fasting blood glucose, mg/dL	100.5 ± 20.4	108.5 ± 32.0	112.2 ± 35.3	0.045*
Triglyceride, mg/dL	114.3 ± 82.2	118.4 ± 77.1	99.2 ± 40.8	0.737
Total cholesterol, mg/dL	170.0 ± 30.7	173.7 ± 35.7	170.9 ± 40.3	0.664
LDL, mg/dL	96.5 ± 30.6	104.3 ± 29.3	102.0 ± 39.7	0.590
HDL, mg/dL	45.6 ± 12.3	44.3 ± 11.5	46.3 ± 10.6	0.764

**NOTE.** Data is shown by mean ± standard error or case number (proportion).

rs10846744 GG genotype is significantly associated with a higher fasting blood glucose levels than CC genotype.

**Abbreviations:** HDL: high-density lipoprotein; LDL: low-density lipoprotein.

^*^Wilcoxon rank-sum test.

**Table 3:** Associations of serum HCV titers and SCARB1 genotypes in 156 chronic hepatitis C patients.

	rs10846744			*P* value
	CC genotype	CG genotype	GG genotype	
HCV titers	6.10 ± 0.98	5.66 ± 1.07	5.68 ± 1.05	0.0349*
				0.0415#
	**rs5888**			
	**GG genotype**	**AG genotype**	**AA genotype**	
HCV titers	5.90 ± 0.97	5.64 ± 1.17	6.22 ± 0.82	0.2610
				0.1675
	**rs3782287**			
	**GG genotype**	**AG genotype**	**AA genotype**	
HCV titers	5.78 ± 1.05	5.95 ± 1.08	5.74 ± 0.57	0.3466
				0.6366

**NOTE.** Data is shown by mean ± standard error.

rs10846744 CC genotype is significantly associated with a higher serum HCV titers.

^*^Kruskal-Wallis test.

^#^ANOVA.

**Table 4:** Associations of virological responses with SCARB1 genotypes in 156 chronic hepatitis C patients receiving pegylated interferon plus ribavirin therapy.

Virological response, n(%)	rs10846744			*P* value
	CC genotype	CG genotype	GG genotype	
SVR	42(71.2)	61(84.7)	15(60.0)	0.028
Non-SVR	17(28.8)	11(15.3)	10(40.0)	
cEVR/pEVR	49/4(90.7/7.4)	65/4(92.9/5.7)	20/2(83.3/8.3)	0.435
Non-EVR	1(1.9)	1(1.4)	2(8.3)	
RVR	38(64.4)	55(76.4)	17(68.0)	0.312
Non-RVR	21(35.6)	17(23.6)	8(32.0)	
EOTR	56(96.6)	71(98.6)	23(95.8)	0.665
Non-EOTR	2(3.5)	1(1.4)	1(4.2)	
	**rs10846744**			
rs8099917 GG/GT genotype	**CC & CG genotype**	**GG genotype**		
SVR	8(72.7)		0(0)	0.007
Non-SVR	3(27.3)		5(100)	
	**rs5888**			
	**GG genotype**	**AG genotype**	**AA genotype**	
SVR	65(75.6)	44(74.6)	9(90.0)	0.562
Non-SVR	21(24.4)	15(25.4)	1(10.0)	
cEVR/pEVR	77/4(91.7/4.8)	49/4(92.5/7.6)	8/1(80.0/10.0)	0.350
Non-EVR	3(3.6)	0(0)	1(10.0)	
RVR	61(70.9)	40(67.8)	9(90.0)	0.359
Non-RVR	25(29.1)	19(32.2)	1(10.0)	
EOTR	82(96.5)	57(98.3)	10(100.0)	0.695
Non-EOTR	3(3.5)	1(1.7)	0(0)	
	**rs3782287**			
	**GG genotype**	**AG genotype**	**AA genotype**	
SVR	79(76.7)	36(75.0)	2(50)	0.474
Non-SVR	24(23.3)	12(25.0)	2(50)	
cEVR/pEVR	90/5(91.8/5.1)	39/5(86.7/11.1)	4/0(100/0)	0.692
Non-EVR	3(3.1)	1(2.2)	0(0)	
RVR	77(74.8)	29(60.4)	3(75.0)	0.195
Non-RVR	26(25.2)	19(39.6)	1(25.0)	
EOTR	99(98.0)	46(95.8)	4(100)	0.697
Non-EOTR	2(2.0)	2(4.2)	0(0)	

**NOTE**. This table showed the associations of virological response and SCARB1 genotypes. Use virological response (such as SVR, RVR) as the dependent variable, SCARB1 genotypes as independent variables.

Because rs10846744 CC/CG genotype is significantly associated with a higher SVR rate than GG genotype, the rs10846744 genotype may help in the prediction of SVR among chronic hepatitis C patients with unfavorable IL28B genotype (rs8099917 GG/GT genotype) following pegylated interferon plus ribavirin therapy.

**Abbreviations:** SVR, Sustained virologic response; VR, Virologic response; RVR, rapid virologic response; EVR, early virologic response; cEVR, complete early virologic response; pEVR, partial early virologic response; EOTR, end-of-treatment response.

**Table 5:** Factors associated with SVR identified by multivariate analyses in 156 chronic hepatitis C subjects receiving pegylated interferon plus ribavirin therapy.

Clinical factors	OR(95% CI)	*P* value
Age, year	0.95(0.91–1.004)	0.071
Sex, (Male vs. Female)	5.09(2.09–12.4)	<0.001
HCV RNA Log10, IU/mL	0.39(0.22–0.68)	0.001
ALT,U/L	1.003(0.999–1.007)	0.166
PLT, K/ul	1.003(0.99–1.01)	0.512
rs10846744 genotype, (GG genotype = 1)	0.32(0.11–0.95)	**0.039**

**NOTE.** This table showed the associations of clinical factors and SVR. Use SVR as the dependent variable, and age in 1-year increment, sex, serum HCV RNA, ALT, platelet count, and rs10846744 genotype as independent variables. Serum HCV RNA level is Log 10 transformed.

rs10846744 GG genotype is significantly associated with a lower SVR rate in multivariate analyses.

**Abbreviations:** ALT, alanine aminotransferase; PLT, platelet count.
