# Supplementary material for: Association of SCARB1 Gene Polymorphisms with Virological Response in Chronic Hepatitis C Patients Receiving Pegylated Interferon plus Ribavirin Therapy
Source: Sci Rep. 2016 Aug 26;6:32303. doi: 10.1038/srep32303 (PMC4999819; doi:10.1038/srep32303)
Supplement: Supplementary Information [file srep32303-s1.doc]

**Supplementary Information**

**Title: Association of SCARB1 Gene Polymorphisms with Virological Response in Chronic Hepatitis C Patients Receiving Pegylated Interferon plus Ribavirin Therapy**

Ching-Sheng Hsu^1,2,3^, Shih–Jer Hsu^4,5^, Wei-Liang Liu^5^, Ding-Shinn Chen^5,6^, Jia-Horng Kao*,^5,6,7,8^

^1^Division of Gastroenterology, Department of Internal Medicine, Taipei Tzu Chi Hospital, Buddhist Tzu Chi Medical Foundation, Taipei, Taiwan

^2^School of Post-Baccalaureate Chinese Medicine, Tzu Chi University, Hualien, Taiwan

^3^School of Medicine, Tzu Chi University, Hualien, Taiwan

^4^Department of Internal Medicine, National Taiwan University Hospital, Yun-Lin Branch, Yun-Lin County, Taiwan

^5^Graduate Institute of Clinical Medicine, National Taiwan University College of Medicine, Taipei, Taiwan

^6^Department of Internal Medicine, National Taiwan University College of Medicine and National Taiwan University Hospital, Taipei, Taiwan

^7^Department of Medical Research, National Taiwan University College of Medicine and National Taiwan University Hospital, Taipei, Taiwan

^8^Hepatitis Research Center, National Taiwan University College of Medicine and National Taiwan University Hospital, Taipei, Taiwan

**Supplementary Table S1. Associations of SVR with SCARB1 genotypes in chronic hepatitis C patients receiving pegylated interferon plus ribavirin therapy and stratified by HCV genotype 1 & 2.**

| **Virological response, n(%)** | **rs10846744** | | | **P value** |
| --- | --- | --- | --- | --- |
|  | **CC genotype** | **CG genotype** | **GG genotype** |  |
| **SVR** | 42(71.2) | 61(84.7) | 15(60.0) | 0.028 |
| **Non-SVR** | 17(28.8) | 11(15.3) | 10(40.0) |  |
| **HCV genotype 1** | | | | |
| **SVR** | 31(64.6) | 47(85.5) | 14(60.9) | 0.021 |
| **Non-SVR** | 17(35.4) | 8(14.6) | 9(39.1) |  |
| **HCV genotype 2** |  |  |  |  |
| **SVR** | 8(100.0) | 11(84.6) | 1(50.0) | 0.160 |
| **Non-SVR** | 0(0) | 2(15.4) | 1(50.0) |  |
